# Isolation of* Bacillus sphaericus* from Lombok Island, Indonesia, and Their Toxicity against* Anopheles aconitus*

**DOI:** 10.1155/2015/854709

**Published:** 2015-12-14

**Authors:** Bambang Fajar Suryadi, Bagyo Yanuwiadi, Tri Ardyati, Suharjono

**Affiliations:** ^1^Department of Biology, Faculty of Mathematics and Natural Sciences, Mataram University, Mataram 83125, Indonesia; ^2^Department of Biology, Faculty of Mathematics and Natural Sciences, Brawijaya University, Malang 65145, Indonesia

## Abstract

Malaria is endemic to Lombok Island, Indonesia. One approach to suppress malaria spread is to eliminate anopheline larvae in their habitat and the environmentally safe agent is bacteria, that is,* Bacillus sphaericus*. However, there is no information regarding local isolate of* B. sphaericus* that is toxic to mosquito larvae from Lombok. The aims of the study were to isolate* B. sphaericus* from soil in areas close to beach surrounding Lombok Island and to test their toxicity against 3rd instar* Anopheles aconitus* larvae. Soil samples were collected from 20 different sampling locations from Lombok Island and homogenized with sterile physiological salt solution. Suspension was heat-shocked at 80°C for 30 minutes and then spread onto antibiotic-supplemented NYSM solid medium. Colonies grown were characterized and subjected to initial toxicity test against anopheline larvae. Isolates with more than 50% killing percentage were subjected to bioassay testing against anopheline larvae. From 20 locations, 1 isolate showed mild toxicity (namely, isolate MNT) and 2 isolates showed high toxicity (namely, isolates SLG and TJL2) against* An. aconitus*. Those 3 isolates were potentially useful isolates, as they killed almost all larvae in 24 hours. The discovery of toxic indigenous isolates of* B. sphaericus* from Lombok Island opens opportunity to develop a biopesticide from local resources.

## 1. Introduction

Lombok Island is one island in West Nusa Tenggara Province of Indonesia. One of common infectious diseases on Lombok is malaria. It is predicted that at minimum 13,000 people are suffering from the disease [1]. Malaria is caused by a protozoa called* Plasmodium* and spreads among humans by bites from anopheline mosquitoes. One species that has been identified as a malarial vector on Lombok is* Anopheles aconitus*. The species lives at locations ranging from sea level to 600–800 m above sea level. The larvae of* An. aconitus* can be found on rice fields (planted and unplanted), various shallow pools (rock, stream, and flood), and slow moving streams with grassy margins [2].

Mosquito control is the primary method used to suppress the spread of malaria. This is commonly done in 3 ways: mosquito larvae control (using larvicide), adult mosquito control (using adulticide), and breeding habitat modification [3]. The most effective approach is mosquito larvae control and this can be accomplished in several ways. One safe agent for controlling anopheline larvae is bacteria called* Bacillus sphaericus* [4].

The use of indigenous* B. sphaericus* is highly desirable as it would build a local capability to produce a biopesticide in developing countries. The capability would suppress dependency on imported product and accelerate biopesticide production [5]. However, there is no information on prevalence of environmentally relevant* B. sphaericus* on Lombok nor its potential to be used as a biopesticide. Therefore, studies to reveal indigenous isolate of* B. sphaericus* are important to the island from both a public health and economic perspectives.

In this study, isolates of* B. sphaericus* were taken from some areas close to beach area and villages known to be endemic to malaria. The isolates were tested against* An. aconitus* larvae that is widely found on the island.

## 2. Material and Methods

### 2.1. Soil Collection

Collection was done at 20 different locations close to beach area surrounding Lombok Island, West Nusa Tenggara, Indonesia. Five hundred grams of soil was collected from each chosen point compositely and stored in sterile screw-capped container. The chosen areas were close to village and/or river opening/estuaries presumably an appropriate location for anopheline breeding habitat.

### 2.2. Bacterial Isolation

Soil samples were homogenized with sterile physiological salt solution forming 10% w/v suspension. The suspension was heated to 80°C for 30 minutes and then serially diluted with sterile physiological saline solution (in 10^−1^ to 10^−5^ dilutions). Diluted suspension was spread on NYSM (nutrient agar enriched with 0.5 g/L yeast extract, 0.2 g/L MgCl_2_, 0.01 g/L MnCl_2_, and 0.1 g/L CaCl_2_) plating medium supplemented with 100 *μ*g/mL streptomycin to avoid unwanted bacteria growth [6]. Incubation was done at 30°C for 2 × 24 hours. Colonies that showed Gram positive rod with bulging endospore on the terminal end were purified for detailed characterization and toxicity testing. Putative* B. sphaericus* isolates were further characterized using key biochemical/physiological tests such as catalase, oxidase, nitrate reduction, urease, sugar utilization, starch hydrolysis, and antibiotic sensitivity test [7].

### 2.3. Larvae Preparation

Anopheline eggs came from mosquitoes reared intensively in rearing facility at IVRCRD (Institute for Vector and Reservoir Control Research and Development), Salatiga, Central Java, Indonesia. Anopheline eggs were submerged into well water (nontreated water) to hatch them. Larvae resulting from hatched eggs were reared for 6 days to reach 3rd instar stadium (3-4 mm in length).

### 2.4. Initial Toxicity Testing

This testing is done to observe toxicity potential of all* B. sphaericus* isolates. The procedure was described by Dulmage et al. [8]. The* B. sphaericus* isolates were grown in NYSM liquid medium at 30°C for 72 hours with 170 rpm shaking. Sixty anopheline larvae (60 larvae in 3 containers) were put into 10% v/v isolated* B. sphaericus* grown on the NYSM liquid medium. Larva death on each test replication was observed and mean value of larva death was calculated. The test was also done with* B. sphaericus* 2362 for comparison.

### 2.5. Bioassay

In order to obtain LC (lethal concentration) value, bioassay was done on isolates that showed more than 50% toxicity on initial toxicity testing. As mention by Dulmage et al. [8], seven concentrations (in 10-fold concentration differences with 3 replications) of chosen isolated* B. sphaericus* grown in 3 × 24-hour NYSM liquid medium were prepared (totally there were 21 testing containers). Four hundred and twenty* An. aconitus* larvae were distributed evenly in the container (20 larvae for each testing container). Sixty* An. aconitus* larvae in 3 containers (20 larvae per testing container) were mixed with 10% v/v culture medium (without bacteria) as negative control and other 60* An. aconitus* larvae were mixed with culture of* B. sphaericus* 2362 as positive control.

Mortality rate of anopheline larvae was calculated using this formula:

(1) $$\begin{array}{c} \text{Mortality rate}=\frac{\text{number of dead larvae}}{\text{number of total larvae}}\times 100\%. \end{array}$$

If in the negative control group 5–20% dead larva are found, Abbott's correction formula [9] is used to obtain corrected mortality rate:

(2) Corrected  mortality  rate=Mortality  rate  of  test  group−mortality  rate  of  control  group100%−Mortality  rate  of  control  group×100%.

Lethal concentration (LC) values in 24 and 48 hours were calculated using Probit analysis [10] applying software Minitab V16 for Windows.

## 3. Results and Discussion

The use of* B. sphaericus* as a biocontrol agent to suppress anopheline larvae has been done intensively in some countries, such as United States, some European countries, and China. It is primarily used at standing waters, swamps/marshland, paddy fields, and lake shores that are known as primary mosquito breeding habitats [11–13].

**Figure 1:**
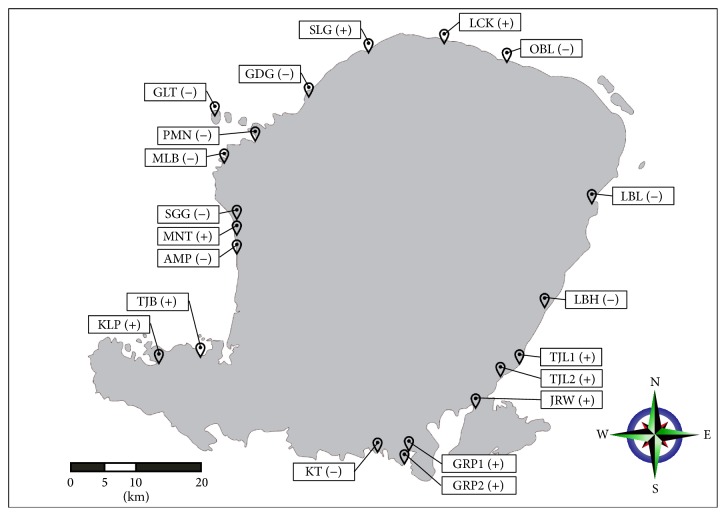
Sampling location of* B. sphaericus* around Lombok Island (Indonesia) and their toxicity attributes ((−): nontoxic; (+): toxic).

In this study 20 isolates of* B. sphaericus* (and their toxicity attributes) were found at 20 locations close to beach area around Lombok Island Indonesia as presented in Figure 1.

**Figure 2:**
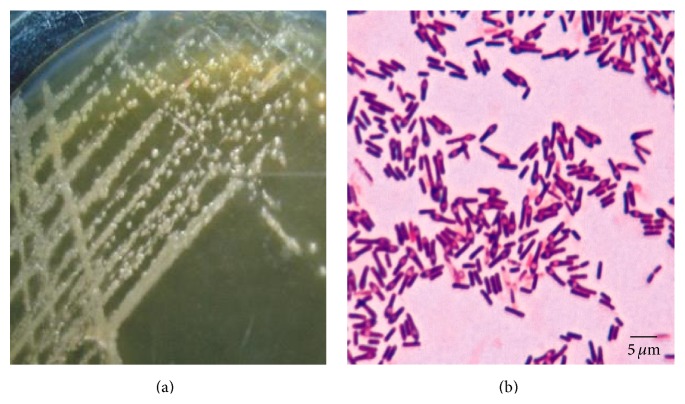
Morphology of colony (a) and cell (b) of* B. sphaericus* from Lombok Island in NYSM agar medium on 3 × 24-hour incubation.

Morphology characteristics of* B. sphaericus* isolated from Lombok Island are presented in Figure 2 and their characteristics are presented on Table 1.

The characteristics of putative* B. sphaericus* isolates were in agreement with standard characteristics mentioned in* Bergey's Manual of Determinative Bacteriology* [7].

We have found that all these isolates could be not collected from area exposed to sea water directly (in form of rip-tide or salt dam/pool).* B. sphaericus* isolates were isolated from sand/soil covered/shaded with leaves and rich of organic matter (grass, fallen leaves, branches, etc.). Some locations formed small puddles, while other locations were dry and/or moist soil.

From 20 locations explored, medium and highly toxic* B. sphaericus* was obtained only from 3 locations. Compared to other locations, these 3 locations were areas that formed small puddles that were rich in organic matter, shaded from the sun, undisturbed by human activity, and inhabited by mosquito larvae, whereas other locations were dry or moist areas (and no mosquito larvae was found). However, the latter were also rich in organic matter, shaded, and untouched. These locations were in accordance with the first discovery of* B. thuringiensis* in Israel Nagev Dessert [14] and discovery of* B. sphaericus* in the United States [15]. Those reports had similarities that those entomopathogenic bacteria were isolated from small puddle inhabited by mosquito larvae.

Soil is potential habitat for* Bacillus*, as soil provides nutrients and growing factors for the bacteria. However, in this study richness in organic matter did not make a given area a suitable habitat for toxic* B. sphaericus*. It was seen that, from 20 locations explored, there were 10 locations that gave nontoxic* B. sphaericus* isolates, 7 locations that gave lowly toxic* B. sphaericus* isolates, and only 3 locations that gave very toxic* B. sphaericus* isolates. We suggest that richness in organic matter is not main factor for obtaining such toxic* B. sphaericus* isolates. Contact with mosquito larvae should be taken into consideration and it was shown in some report in early discovery of entomopathogenic bacteria [14, 15].

LC_50_ and LC_90_ values in 24 and 48 hours of three* B. sphaericus* isolates based on cell concentration (cell/mL) are shown in Table 2. Isolate MNT showed higher LC values compared to* B. sphaericus* 2362 as standard. Isolates SLG and TJL2 showed LC values that were close to those of* B. sphaericus* 2362. LC (lethal concentration) value informs us how low the concentration or the dilution of certain microbe or ingredient able to kill targeted organism is. From these values it can be concluded that* B. sphaericus* isolate MNT was of lower toxicity than* B. sphaericus* 2362, whereas* B. sphaericus* isolates SLG and TJL2 had LC values that were almost similar to toxicity of* B. sphaericus* 2362.

These* B. sphaericus* isolates were the first toxic* B. sphaericus* isolated from Lombok Island, Indonesia. Other entomopathogenic bacterium that was isolated and tested was* B. thuringiensis* that came from some areas in Indonesia.* B. thuringiensis'* susceptible targets are larvae* Aedes* and* Culex*.* Anopheles* is the least susceptible to this bacterium. In contrast,* B. sphaericus*' susceptible targets are* Culex* and* Anopheles*, whereas* Aedes* is the least susceptible. The toxicity and LC value of this new isolated* B. sphaericus* suggest that it would be good candidate for local biocontrol agent on Lombok Island.

*B. sphaericus* can kill mosquito larvae because of toxin activities it harbors. There are 2 kinds of toxins: binary toxins/Bin (51 and 42 kDa) are produced on sporulating stage and mosquitocidal toxins/Btx (100, 32 and 36 kDa) are produced on vegetative stage [16, 17]. The binary toxins which are the most potent toxins can interact with receptor along larvae midgut specifically, whereas the mosquitocidal toxins are weaker toxins that will kill the larvae in longer period or will not kill at all (just weaken the larvae) [18]. The activities of the toxins cause nervous and muscle system collapse of the larvae. The larvae will lose its ability to move and consequently undergo asphyxia by drowning [19]. The existence of the toxins varies. Some strains may have both toxins; others may have only one or none. That explains varied killing capability among strains of* B. sphaericus* worldwide [20]. From its low toxicity and higher LC values (compared to* B. sphaericus* 2362), we predict that isolate MNT may only have binary toxins, while other 2 isolates may have binary toxin and mosquitocidal toxin altogether.

Compared to other biocontrol bacteria such as* B. thuringiensis*,* B. sphaericus* will last longer in environment (some study reported 20–30 days after application) [21]. Also,* B. sphaericus* is still effective in killing mosquito larvae on polluted waters [22]. These reasons make* B. sphaericus* a popular biocontrol agent in some countries.

Even though many* B. sphaericus* strains from any places in the world have been collected, the existence of indigenous isolates is still important to study, as it will open opportunity to develop local-strain-based biopesticide production in developing countries such as Indonesia. This capability will suppress cost used for importing commercial biopesticide from other countries and also promote local biopesticide industry as well.

## 4. Conclusion

Twenty local isolates of* B. sphaericus* were found from 20 locations close to beach area on Lombok Island with varied toxicity against anopheline larvae. Isolate MNT was mildly toxic against* An. aconitus* larvae, while isolates SLG and TJL2 were highly toxic against* An. aconitus*.

## Figures and Tables

**Table 1:** Characteristics of *B. sphaericus* isolated from Lombok Island.

Characteristics	Results/isolated	Standard
Cell		
Form	Rod	Rod
Gram reaction	Positive	Positive
Size (*L* × *W*)	3.0–5.0 × 0.5–0.75 *μ*m	1.5–5.0 × 0.6–1.0 *μ*m
Endospore	Positive	Positive
Endospore position	Terminal	Terminal/subterminal
Bulging endosporangium	Positive	Positive
Morphology		
Form	Round	Round
Margin	Entire	Entire
Surface	Flat and smooth	Flat and smooth
Color	White-cream	Opaque (grown on nutrient agar)
Biochemical and physiological		
Catalase	Positive	Positive
Starch hydrolysis	Negative	Negative
Acid production from sugar	Negative	Negative
Nitrate reduction	Negative	Negative
Urease	Positive	Positive
Oxidase	Positive	Positive
Aerobicity	Aerobe	Aerobe
Sensitivity to streptomycin	Resistant	Resistant
Sensitivity to chloramphenicol	Sensitive	Sensitive
Sensitivity to penicillin	Sensitive	Sensitive
Sensitivity to tetracycline	Sensitive	Sensitive
Sensitivity to amoxicillin	Sensitive	Sensitive
Sensitivity to vancomycin	Sensitive	Sensitive
Sensitivity to erythromycin	Sensitive	Sensitive
Sensitivity to gentamicin	Sensitive	Sensitive
Sensitivity to ciprofloxacin	Sensitive	Sensitive

**Table 2:** LC value of *B. sphaericus* isolated from Lombok Island against *An. aconitus* and its comparison with *B. sphaericus* 2362.

Isolates	LC values (cell/mL)			
	LC_50-24 hrs_	LC_90-24 hrs_	LC_50-48 hrs_	LC_90-48 hrs_
*B. sphaericus* isolate MNT	1.28 × 10^8^	1.76 × 10^7^	1.98 × 10^8^	4.57 × 10^7^
*B. sphaericus *isolate SLG	1.51 × 10^7^	3.69 × 10^6^	2.54 × 10^5^	5.45 × 10^5^
*B. sphaericus* isolate TJL2	1.12 × 10^6^	4.33 × 10^6^	1.01 × 10^5^	4.25 × 10^5^
*B. sphaericus* 2362 (standard)	1.52 × 10^6^	1.34 × 10^5^	5.64 × 10^6^	5.88 × 10^5^
